# Decoding Acute Myeloid Leukemia: A Clinician’s Guide to Functional Profiling

**DOI:** 10.3390/diagnostics14222560

**Published:** 2024-11-14

**Authors:** Prasad Iyer, Shaista Shabbir Jasdanwala, Yuhan Wang, Karanpreet Bhatia, Shruti Bhatt

**Affiliations:** 1 Children’s Blood and Cancer Centre, KK Women’s and Children’s Hospital, Singapore 229899, Singapore; 2 Duke-NUS Medical School, Singapore 169857, Singapore; 3 Department of Pharmacy, National University of Singapore, Singapore 119077, Singapore; shaista@nus.edu.sg (S.S.J.); e1124510@u.nus.edu (Y.W.); shruti_bhatt@nus.edu.sg (S.B.); 4 Department of Hematology and Medical Oncology, School of Medicine, Winship Cancer Institute, Emory University, Atlanta, GA 30322, USA; karanpreet.bhatia@emory.edu

**Keywords:** acute myeloid leukemia, functional profiling, BH3 profiling, gene expression profiling, proteomics, metabolomics, drug sensitivity/resistance testing

## Abstract

Acute myeloid leukemia (AML) is a complex clonal disorder characterized by clinical, genetic, metabolomic, and epigenetic heterogeneity resulting in the uncontrolled proliferation of aberrant blood-forming precursor cells. Despite advancements in the understanding of the genetic, metabolic, and epigenetic landscape of AML, it remains a significant therapeutic challenge. Functional profiling techniques, such as BH3 profiling (BP), gene expression profiling (GEP), proteomics, metabolomics, drug sensitivity/resistance testing (DSRT), CRISPR/Cas9, and RNAi screens offer valuable insights into the functional behavior of leukemia cells. BP evaluates the mitochondrial response to pro-apoptotic BH3 peptides, determining a cell’s apoptotic threshold and its reliance on specific anti-apoptotic proteins. This knowledge can pinpoint vulnerabilities in the mitochondria-mediated apoptotic pathway in leukemia cells, potentially informing treatment strategies and predicting therapeutic responses. GEP, particularly RNA sequencing, evaluates the transcriptomic landscape and identifies gene expression alterations specific to AML subtypes. Proteomics and metabolomics, utilizing mass spectrometry and nuclear magnetic resonance (NMR), provide a detailed view of the active proteins and metabolic pathways in leukemia cells. DSRT involves exposing leukemia cells to a panel of chemotherapeutic and targeted agents to assess their sensitivity or resistance profiles and potentially guide personalized treatment strategies. CRISPR/Cas9 and RNAi screens enable systematic disruption of genes to ascertain their roles in leukemia cell survival and proliferation. These techniques facilitate precise disease subtyping, uncover novel biomarkers and therapeutic targets, and provide a deeper understanding of drug-resistance mechanisms. Recent studies utilizing functional profiling have identified specific mutations and gene signatures associated with aggressive AML subtypes, aberrant signaling pathways, and potential opportunities for drug repurposing. The integration of multi-omics approaches, advances in single-cell sequencing, and artificial intelligence is expected to refine the precision of functional profiling and ultimately improve patient outcomes in AML. This review highlights the diverse landscape of functional profiling methods and emphasizes their respective advantages and limitations. It highlights select successes in how these methods have further advanced our understanding of AML biology, identifies druggable targets that have improved outcomes, delineates challenges associated with these techniques, and provides a prospective view of the future where these techniques are likely to be increasingly incorporated into the routine care of patients with AML.

## 1. Introduction

Acute myeloid leukemia (AML), the most common leukemia in adults, is a rapidly progressing and aggressive malignancy of the blood and bone marrow that affects myeloid cells. In AML, these cells transform into malignant immature blasts that proliferate within the bone marrow, impeding the production of healthy blood cells. Genetic and epigenetic clonal alterations that disrupt normal cell growth and differentiation are hypothesized to be central to the etiology of AML. Rather than developing into functional cells, these blasts multiply uncontrollably, resulting in a range of symptoms including severe fatigue, abnormal bruising, recurrent infections, and anemia. While recent advancements in chemotherapy and enhanced supportive care have resulted in improved survival outcomes, these gains are predominantly observed in younger patients and those with a favorable risk of disease; nevertheless, the cure rates remain suboptimal [1]. Allogeneic hematopoietic stem cell transplantation (HSCT) remains the sole curative intervention for patients with intermediate or high-risk disease [2]. However, a significant proportion of AML patients are ineligible for this treatment modality because of their advanced age and comorbidities, thus contributing to the poor survival rates observed in this population [3]. A more comprehensive understanding of AML biology remains an unmet need for the development of novel, precise, and tolerable therapeutic approaches. Recent advancements in functional profiling methodologies offer the potential to enhance our understanding of and refine approaches that are crucial for improved disease stratification. These methodologies will help us identify potential therapeutic targets with the aim of improving survival rates and quality of life for all patients with AML. In this brief narrative review, we discuss recent updates in functional profiling techniques, explore the advantages and disadvantages of different methods, and discuss the potential integration of these methods in clinical practice.

## 2. Functional Profiling in Oncology

Functional profiling involves the evaluation of the activity of various cellular pathways, gene expression, and biochemical responses to identify specific vulnerabilities and treatment targets. This includes genomic, transcriptomic, proteomic, and metabolomic profiling. Genomic profiling identifies DNA mutations or alterations, whereas transcriptomic profiling examines RNA transcripts to identify gene expression patterns and regulatory mechanisms. Proteomic profiling studies protein expression and modifications to demonstrate the effects of genomic and transcriptomic changes. Metabolomic profiling is used to assess metabolite concentrations and fluxes, indicating cellular biochemical activity.

In cancer research, functional profiling offers significant benefits by identifying biomarkers for early detection, prognosis, and therapeutic response, thereby potentially enhancing personalized medicine. It helps to uncover the molecular mechanisms of tumorigenesis and metastasis to identify novel therapeutic targets and pathways. Additionally, it aids in understanding drug resistance mechanisms and in guiding the development of combination therapies.

In summary, these new techniques offer a comprehensive method for understanding complex cellular processes, significantly enhancing cancer diagnosis and treatment, and understanding oncogenic mechanisms, thus enabling precision therapy.

## 3. Functional Profiling Techniques

### 3.1. Gene Expression Profiling (GEP)

Contemporary methodologies for GEP in AML include RNA sequencing, microarray analysis, and quantitative PCR, all of which provide distinct insights into the molecular architecture of the disease. Table 1 summarizes key advantages and disadvantages of the different available techniques. Recent advancements in AML research have provided substantial insights into the molecular mechanisms underlying this disease. High-throughput sequencing technologies have facilitated comprehensive analyses, revealing distinct gene expression patterns that correlate with specific genetic mutations and cytogenetic abnormalities [4,5]. These findings have elucidated the pathways critical to leukemogenesis, such as aberrant signaling cascades and disrupted differentiation processes. Recent studies have identified several genes that are overexpressed in AML, including *STMN1*, *KITLG*, *CDK6*, *MCM5*, *KRAS*, *CEBPA*, *MYC*, *ANGPT1*, *SRGN*, *RPLP0*, *ENO1*, and *SET* [6]. A separate investigation identified the top 20 mutated genes in AML as *PIFC*, *DNMT3A*, *TTN*, *NPM1*, *RUNX1*, *TP53*, *IDH2*, *FLT3*, *HPS3*, *SENP6*, *ABCA6*, *ASXL1*, *BRWD1*, *DNAH11*, *GABRG3*, *GDI2*, *KRAS*, *MUC16*, *PCLO*, and *SLIT2*. The study demonstrated that elevated expression of FLT3, ABCA6, and PCLO was associated with unfavorable prognosis, whereas increased expression of SLIT3 and HSP3 was correlated with improved patient outcomes [7]. These genes appear to be integral to AML pathogenesis. GEP enables the prediction and categorization of AML into various subgroups, encompassing both genetically defined categories and newly identified clusters that correlate with the outcomes [8,9,10,11,12].

GEP revealed mechanisms of resistance to venetoclax and azacitidine linked to the intrinsic properties of monocytic differentiation, and highlighted an inherent dependence on Mcl-1, suggesting therapeutic targeting in the future [13]. GEP also highlighted *FLT3*-like gene expression in some wild-type *FLT3* AML samples for which FLT3 inhibitors could be considered [14]. Another recent study identified dasatinib as a drug that targets AML blasts and LSCs in a subset of patients with AML via GEP [15].

This expanding body of literature enhances our understanding of the molecular mechanisms underlying AML. It also underscores the potential of personalized medical approaches and the optimization of treatment strategies based on individual molecular profiles.

### 3.2. Proteomics and Metabolomics

Recent advancements in high-throughput technologies, particularly proteomics and metabolomics, have demonstrated their significant importance in our understanding of AML. Proteomics encompasses a comprehensive examination of protein expression, post-translational modifications, and interactions within cells. This methodology plays a crucial role in identifying aberrant signaling pathways and potential therapeutic targets in AML. For instance, proteomic analysis can reveal dysregulated kinases and altered protein networks that contribute to leukemogenesis [16]. Ongoing proteomic research, including the examination of post-translational modifications, has played a crucial role in discovering biomarker proteins vital for leukemic cell persistence, evaluating treatment effectiveness, and further categorizing AML [17,18,19]. Emerging evidence suggests a potential role for proteomics in investigating post-HSCT relapses within the context of MDS, wherein methylation changes and rejection pathways appear to be implicated [20]. Metabolomics investigates small-molecule metabolites and their fluxes, providing insights into the altered metabolic states and pathways in neoplastic cells. Metabolomic research on AML has elucidated specific metabolic signatures, including enhanced glycolysis, altered glucose metabolic profiles, distinctive variations in lipid composition among different AML cells, and impaired mitochondrial function, which facilitate the survival and proliferation of leukemia cells [21,22,23,24,25,26,27]. The metabolomic profiles obtained in a recent study revealed numerous alterations in glycerophospholipid and sphingolipid metabolisms [28]. These findings facilitated the identification of potential biomarkers that could aid in diagnosis and subsequent classification. This study emphasizes the utility of liquid chromatography coupled with mass spectrometry (LC–MS)-based metabolomics on serum samples as a diagnostic tool and a potentially minimally invasive prognostic method. This approach may prove beneficial in identifying various cytogenetic changes and prognosticating treatment outcomes in patients with AML. By integrating proteomic and metabolomic data, researchers can obtain a more comprehensive understanding of AML and facilitate the identification of novel biomarkers for its diagnosis, prognosis, and future therapeutic interventions [29]. Furthermore, these approaches enable the investigation of drug resistance mechanisms and are essential tools for ongoing efforts to elucidate the molecular foundations of AML.

### 3.3. Drug Sensitivity/Resistance Testing (DSRT), CRISPR/Cas9, and RNAi

Drug sensitivity and resistance testing in AML is likely to be routinely integrated to optimize treatment strategies. Given that AML is a heterogeneous and aggressive malignancy, patient responses to chemotherapy vary significantly. Drug sensitivity assays involve exposing leukemia cells to various chemotherapeutic agents and evaluating cell viability to determine the most efficacious drugs. Resistance testing aims to identify ineffective drugs and enables oncologists to avoid futile treatments.

Multiple methodologies are employed in these assays. Conventional techniques measure metabolic activity to infer cell viability after drug exposure. The Cell Titer-Glo (CTG) assay, based on ATP quantification, offers high sensitivity and luminescent readouts, facilitating the expeditious assessment of cell viability with a broader dynamic range [30,31]. Flow cytometry and fluorescence-activated cell sorting (FACS) provide detailed analysis of cell proliferation, apoptosis, and surface marker expression [32]. High-throughput screening (HTS) enables the rapid and simultaneous evaluation of numerous drugs and their combinations, significantly expediting the testing process [33]. Next-generation sequencing (NGS) offers genetic insights by identifying mutations and gene expression profiles associated with drug resistance, particularly when combined with multi-omics and HTS [34]. CRISPR-based screens and RNA interference (RNAi) gene expression silencing techniques can be used to identify genetic dependencies that can be targeted to overcome resistance [34,35,36,37,38]. Table 2 delineates the principal advantages and disadvantages of various RNAi methodologies.

Currently, these drug sensitivity testing methods have demonstrated predictive value and correlation with responses in clinical trials [39,40,41]. The integration of these diverse methodologies with artificial intelligence techniques into clinical practice will gain further traction as they offer the potential for the development of personalized therapeutic regimens aimed at improving outcomes while reducing toxicity.

### 3.4. BH3 Profiling and Dynamic BH3 Profiling

BH3 profiling (BP) and dynamic BH3 profiling (DBP) are novel techniques that provide critical insights into the regulation of apoptosis in leukemic cells and their response to therapeutic interventions [42,43]. BP is a mitochondrial functional assay that quantifies the apoptotic priming, or the propensity of a cell to undergo apoptosis, by measuring its dependence on anti-apoptotic proteins such as Bcl-2, Bcl-X_L_, and Mcl-1 [44]. The methodology involves exposure of tumor cells to synthetic BH3 domain peptides, which simulate pro-apoptotic BH3-only proteins. These peptides interact with anti-apoptotic members of the Bcl-2 family, resulting in mitochondrial outer membrane permeabilization (MOMP). The extent of cytochrome c release following MOMP serves as an indicator of the apoptotic threshold of the cell. This information is essential for elucidating cellular susceptibility to apoptosis and for informing the application of targeted therapies such as Bcl-2 inhibitors (e.g., venetoclax), which are designed to induce apoptosis in AML cells exhibiting high levels of apoptotic priming and dependence on the Bcl-2 protein [45].

Dynamic BH3 profiling (DBP) expands on this methodology by evaluating alterations in apoptotic priming in response to therapeutic agents over time. This technique involves the treatment of AML cells with pharmacological agents and the subsequent implementation of BP to monitor changes in mitochondrial apoptotic sensitivity. Through the measurement of priming modifications in response to treatment, DBP can be utilized to predict the efficacy of therapeutic agents and elucidate potential resistance mechanisms [43,46]. DBP is particularly valuable because it provides a dynamic perspective on the adaptation of leukemia cells to therapeutic interventions. This real-time assessment facilitates the optimization of treatment regimens and the early detection of resistance, enabling timely modifications in therapeutic strategies. For instance, DBP can identify patients who are likely to develop resistance to single-agent therapies, thereby indicating the need for combination therapies to achieve improved clinical outcomes.

Together, BP and DBP represent powerful tools in precision oncology for AML, offering a means to personalize treatment plans based on the apoptotic landscape of individual patient leukemic cells. These approaches enhance our understanding of disease biology and improve the potential for successful therapeutic interventions.

### 3.5. Cytokine Profiling

Cytokine profiling is a crucial technique for elucidating the dynamic interactions between leukemia cells and their microenvironment. Cytokines are essential regulatory molecules that modulate immune responses, inflammation, and hematopoiesis. In AML, analysis of cytokine levels provides critical insights into the pathophysiology of the disease and potential therapeutic interventions [47].

**Figure 1:**
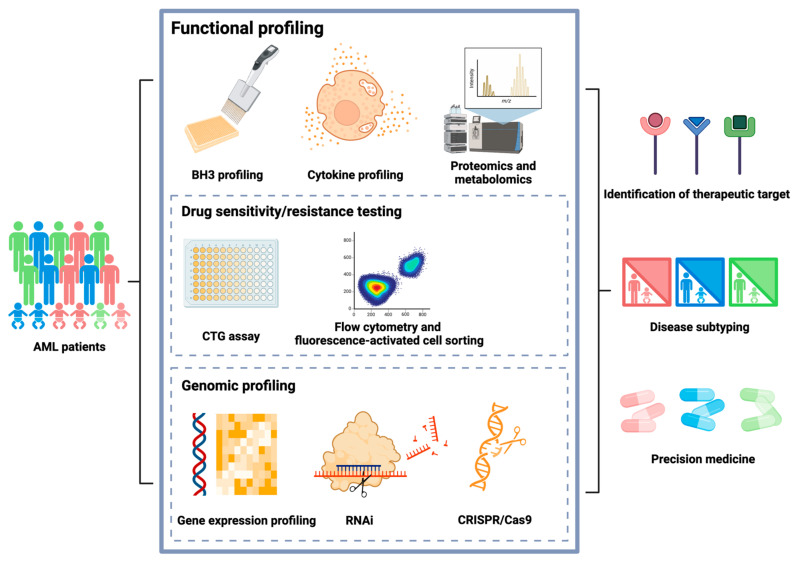
Overview of different types of functional profiling methods and their utility in target identification, disease subtyping, and potential to improve precision medicine.

For instance, elevated levels of interleukin-6 (IL-6) and tumor necrosis factor-alpha (TNF-α) are frequently observed in AML patients [48,49,50,51]. These cytokines are known to promote leukemia cell survival, proliferation, and resistance to apoptosis, thereby contributing to disease progression and poor prognosis. Furthermore, cytokine profiling has been instrumental in elucidating the mechanisms underlying the interaction between AML cells and the bone marrow microenvironment. For instance, elevated levels of vascular endothelial growth factor (VEGF) in the bone marrow plasma of patients with AML have been associated with increased angiogenesis, which facilitates leukemia cell survival and proliferation [52,53]. Targeting cytokine-mediated pathways can disrupt supportive interactions and offer new therapeutic strategies. Cytokine profiling holds substantial promise for advancing our understanding of biological processes and for developing therapeutic approaches that may improve clinical outcomes. Figure 1 presents an overview of the applications of functional profiling in AML.

## 4. Comparative Analysis of Different Techniques

Each functional profiling method has certain advantages that balance predictive accuracy, understanding of mechanisms, and high-throughput capacity against limitations such as the need for viable cells, accessibility, complexity, technical requirements, and cost. Table 3 presents a summary of the principal advantages and disadvantages.

## 5. Applications of Functional Profiling in AML

### 5.1. Diagnosis and Subtyping

Flow cytometry is essential for profiling AML, facilitating comprehensive analysis of cellular populations through assessment of cell dimensions, granularity, and surface and intracellular markers. In AML, it identifies distinctive immunophenotypes of malignant cells, thereby aiding in diagnosis and classification. It detects minimal residual disease post-treatment, providing insights into therapeutic efficacy and prognosis. Furthermore, it is utilized in drug sensitivity and resistance testing to evaluate leukemia cell responses to chemotherapeutics, examining cell viability and apoptotic markers post-drug exposure. This approach elucidates cellular heterogeneity, drug effectiveness, and resistance mechanisms, thus enhancing personalized treatment strategies. While traditional methods emphasize genetic mutations and chromosomal abnormalities, functional characteristics can reveal nuanced differences in how these genetic factors affect cell behavior.

Augmenting genetic profiling with functional profiling offers a thorough analysis of cellular processes such as protein expression, gene activity, and metabolism. This approach can significantly enhance the diagnosis and subtyping of AMLs. For example, two AML patients with the same genetic mutation may display different drug responses or disease progression due to variations in protein expression or metabolic pathways. Functional profiling can clarify these differences and provide a deeper understanding of this disease.

By analyzing the functional state of leukemia cells, researchers can identify biomarkers specific to AML subtypes, improve diagnostic accuracy, and enable more precise patient categorization for tailored treatment. The following examples suggest future advancements in precision oncology for AML therapy:

Example 1: BH3 Profiling

Functional profiling methodologies such as BP can elucidate protein-level variations or apoptosis thresholds that are not discernible through genetic analysis alone, either directly or indirectly. For instance, irrespective of the underlying genetic alterations, AML dependency on the pro-survival BCL-2 protein pathway can be targeted. This suggests that patients may respond favorably to BCL-2 inhibitors such as venetoclax [45].

Example 2: Metabolic Pathway Analysis

Functional profiling of metabolic pathways can be used to identify AML subtypes that rely on mitochondrial metabolism. When functional assays show increased oxidative phosphorylation in some AML cells, this metabolic signature aids subtype identification. Patients with this profile may benefit from therapies targeting mitochondrial function such as 2-deoxyglucose or other metabolic inhibitors. Although these drugs have only been tested in cell lines, they offer the potential to combine traditional therapies with novel agents in the future [54].

Example 3: Cytokine Secretion Profiles

Functional profiling can be used to elucidate cytokine secretion patterns and offer additional details. For example, specific AML subtypes can be identified by their secretion levels of inflammatory cytokines such as IL-6, which are thought to be linked to chemoresistance. [55]. Higher secretion levels of these cytokines may require a distinct therapeutic approach compared with AML subtypes with lower cytokine activity. These data could guide clinicians to precisely tailor immunotherapy or anti-inflammatory treatments in the future. [47].

Example 4: Drug Sensitivity Testing

Drug sensitivity assays in functional profiling can be used to evaluate AML cellular responses to treatments and to identify subtypes with specific chemotherapy sensitivities. This methodology, incorporating the integration of ex vivo drug testing with either an image-based, single-cell functional precision medicine (pharmacoscopy) approach that examines cell fate following drug exposure or conventional high-throughput drug screening integrated with conventional molecular analysis, has already been successfully implemented in clinical medicine, as previously described [39,40,41].

Example 5: Signaling Pathway Activity

Functional profiling can be used to evaluate signaling pathways essential for AML progression. For example, an analysis may indicate that cells from a particular AML subtype exhibit increased PI3K/AKT pathway activity. Patients with this subtype may be candidates for treatment with PI3K or AKT inhibitor treatment [56].

### 5.2. Therapeutic Target Identification

Functional profiling has facilitated the identification of promising therapeutic targets and interventions. For instance, inhibition of DOT1L, which is essential for maintaining the oncogenic state in *KMT2A*-rearranged (formerly *MLL-r*) AML, has emerged from these profiling studies [57,58]. The development of IDH inhibitors and BH3 mimetics represents a recent example of how functional profiling methods elucidate specific mutations and apoptotic pathways in AML, subsequently leading to successful trials demonstrating the benefits of targeting these pathways. The efficacy of venetoclax in AML has been elucidated through BH3 profiling, which demonstrated the importance of BCL-2 inhibition in specific AML subtypes [45,59]. These advanced profiling techniques have translated laboratory findings into targeted therapies, such as ivosidenib, enasidenib, and venetoclax, providing personalized treatment options that improve patient outcomes by directly addressing the molecular and cellular underpinnings of the disease [60,61,62].

### 5.3. Prognosis and Risk Stratification

Gene expression profiling has revealed several critical biomarkers that provide insights beyond traditional genetic mutations. For instance, the differential expression of *MN1* is associated with poor prognosis and resistance to therapy [63,64]. Another example is the overexpression of the *HOX* gene cluster, particularly *HOXA3-10*, which is correlated with aggressive disease behavior and adverse outcomes [65]. An additional example pertains to the expression of the *EVI1* gene. Elevated *EVI1* expression, as identified by GEP, is associated with a poor prognosis and resistance to conventional therapeutic interventions [66]. These biomarkers, detectable via GEP, facilitate more nuanced risk stratification and guide clinicians in considering alternative or investigational treatments.

### 5.4. Drug Resistance Mechanisms

These modern laboratory methods have significantly advanced our understanding of the mechanisms underlying drug resistance in AML patients. For instance, resistant subpopulations such as leukemia stem cells have been identified, which frequently persist after standard chemotherapy [67]. High-throughput screening has elucidated the association between specific mutations and drug resistance, thereby informing the development of targeted therapies [68]. Epigenetic alterations, including DNA methylation, have been shown to influence drug sensitivity, prompting the investigation of HDAC inhibitors [69]. Moreover, functional profiling could facilitate the design of efficacious drug combinations and enable real-time monitoring of resistance evolution, thus contributing to the pre-emptive management of resistance with the hope of enhancing therapeutic outcomes in patients with AML [70,71].

## 6. Current Challenges and Limitations

### 6.1. Technical and Practical Challenges

Functional profiling has the potential to revolutionize personalized treatment strategies. However, significant technical and practical challenges remain to be overcome. The requirement for a sufficient quantity of viable leukemia cells, the necessity for robust standardization of ex vivo drug exposure for analysis, and the evaluation of drugs outside the host tumor microenvironment raises concerns regarding the efficacy of the tested medicines in patients, presenting significant challenges for the routine implementation of functional profiling. Another technical limitation is the difficulty of data interpretation and integration. Owing to the complexity of data generated from high-throughput techniques such as single-cell sequencing and proteomics, integrating these diverse data streams into a cohesive interpretation framework remains a formidable task [72]. The heterogeneity of AML further complicates these efforts, necessitating sophisticated computational tools and algorithms capable of multifaceted data analysis. Despite these advancements, these tools often face limitations in sensitivity and specificity, which can lead to ambiguous or conflicting results, impeding effective clinical decision-making. Nonetheless, achieving such progress will necessitate joint efforts among doctors, experts in molecular biology, and specialists in bioinformatics [73].

### 6.2. Challenges in Translating to Clinical Practice

Bridging the gap between laboratory discoveries and their therapeutic applications requires overcoming several obstacles. Validation of potential biomarkers or therapeutic targets identified through functional profiling is a protracted and resource-intensive process [64,74,75]. Rigorous clinical trials are required to ensure safety and efficacy. Moreover, the dynamic nature of AML and its propensity to develop resistance to treatment present additional limitations. Personalized treatments derived from functional profiling must be adaptable and responsive to these alterations. Access to advanced profiling technologies is often restricted in many clinical settings, particularly in resource-limited areas, owing to high costs, limited accessibility, and a shortage of specialized personnel to interpret results, hindering widespread implementation. Integration of these advanced techniques into large-scale clinical studies is imperative, and such studies should ensure the inclusion of diverse ethnic populations.

## 7. Future Directions and Conclusions

The landscape of functional profiling in AML is poised for significant advancements with the integration of emerging technologies and multi-omics approaches. The future of functional profiling will likely witness the convergence of several innovative methodologies to enhance our understanding of AML pathogenesis and treatment response. One promising direction is the application of single-cell sequencing in functional assays. This approach will provide a comprehensive understanding of the cellular heterogeneity within AML populations. By characterizing functional states at the single-cell level, researchers can identify subpopulations that exhibit resistance to therapy or that possess unique vulnerabilities, thereby facilitating the development of highly targeted treatments. Moreover, the integration of artificial intelligence (AI) and machine learning (ML) with functional profiling data offers substantial potential [76]. AI algorithms can be used to analyze large datasets to elucidate patterns and predict patient-specific responses to therapies. This can lead to more personalized treatment strategies and improved clinical outcomes by identifying the most efficacious drug combinations for individual patients.

Another important future research direction is the enhancement of real-time and in vivo functional profiling techniques. Current methods, such as DBP, are limited to ex vivo analysis. Developing technologies that allow for in vivo functional assessment will provide more accurate representations of drug responses and resistance mechanisms within the tumor microenvironment [36,77].

Additionally, integration of metabolic and epigenetic profiling with traditional functional assays will provide a holistic view of AML biology. Understanding the interplay between metabolic states, epigenetic modifications, and apoptotic signaling pathways can reveal new therapeutic targets and strategies for overcoming drug resistance [72].

Finally, high-throughput screening platforms will continue to evolve and incorporate more sophisticated and physiologically relevant models, such as organoids and patient-derived xenografts [78]. These models can better simulate the tumor environment and provide predictive insights into treatment efficacy.

In conclusion, the future of functional profiling of AML lies in the integration of advanced technologies, single-cell analyses, AI tools, and comprehensive multi-omics approaches. These innovations will pave the way for precision medicine, offering new hope for improved outcomes with lower toxicity of therapy in AML patients.

## Figures and Tables

**Table 1:** Summary of the various techniques available for GEP, delineating their respective advantages and disadvantages.

GEP Technique	Description	Pros	Cons
Microarray	Measures expression of thousands of genes	High throughput, cost-effective	Limited dynamic range, probe dependence
RNA-seq	Sequencing of RNA to quantitate expression levels	High precision, detects novel transcripts	More expensive, requires complex analysis
qRT-PCR	Quantification of specific RNA sequences	Sensitive, precise quantification	Limited to known targets, lower throughput

**Table 2:** Different methodologies available for RNA interference (RNAi), delineating their respective advantages and disadvantages.

RNAi Technique	Pros	Cons
siRNA (small interfering RNA)	Quick and easy to design Highly specific to target mRNA	Temporary effect Requires transfection methods
shRNA (short hairpin RNA)	Longer-lasting gene silencing Can be integrated into host genome	Risk of insertional mutagenesis More complex to design
miRNA (microRNA)	Can target multiple genes Endogenous regulatory mechanism	Off-target effects Complexity in predicting target genes

**Table 3:** Key advantages and disadvantages of different functional profiling methods in AML.

Tool/Technique	Pros	Cons
Flow Cytometry	High specificity and sensitivity Ability to analyze multiple parameters simultaneously Rapid results	Requires fresh samples Complex data analysis High cost of reagents and equipment
Next-Generation Sequencing (NGS)	Comprehensive genomic profiling Detection of rare mutations High sensitivity	High cost and technical complexity Long turnaround time Requires bioinformatics expertise
Single-Cell RNA Sequencing	Detailed cellular resolution Reveals heterogeneity within cell populations Ability to identify novel gene expressions	High cost and computational demand Technical challenges in data interpretation Requires fresh or properly preserved samples
Drug Sensitivity and Resistance Testing (DSRT)	Directly measures cellular response to treatments Provides functional readout of drug efficacy	Time-consuming and labor-intensive Requires viable cells
Proteomics	High-throughput and simultaneous analysis of many proteins Provides direct measurement of protein levels	Limited by the availability of quality antibodies Lower sensitivity compared to other methods
Metabolomics	Comprehensive profiling of metabolites Insight into cellular metabolic states Potential to identify novel biomarkers Can integrate with other omics data for holistic view	High complexity and variability in metabolite levels Requires advanced instrumentation and expertise High cost and complex data interpretation Sample preparation can affect metabolite stability
CRISPR/Cas9 Screening	Precise gene editing and functional analysis Elucidation of gene function and interaction	Off-target effects and ethical considerations Technical challenges and high costs
BH3 Profiling	Determines mitochondrial dependency on anti-apoptotic proteins Useful in predicting response to BH3 mimetics Rapid and relatively simple to perform Provides insight into the priming status of cells	Primarily provides static information at a single time point May not fully capture dynamic changes in the apoptotic machinery Requires fresh, viable cells for accuracy Limited in predicting in vivo responses
Dynamic BH3 Profiling	Measures real-time changes in apoptotic priming in response to drug treatment in vitro Better predictive value for drug response Can identify early markers of therapeutic efficacy	Technically more complex and time-consuming than standard BH3 profiling Requires sophisticated equipment and expertise May still have limitations in predicting long-term patient outcomes

## Data Availability

No new data were created or analyzed in this study. Data sharing is not applicable to this article.
